# L-Theanine Metabolism in Tea Plants: Biological Functions and Stress Tolerance Mechanisms

**DOI:** 10.3390/plants14030492

**Published:** 2025-02-06

**Authors:** Qianying Wang, Jingbo Yu, Wenchao Lin, Golam Jalal Ahammed, Wenli Wang, Ruihong Ma, Mengyao Shi, Shibei Ge, Ahmed S. Mohamed, Liyuan Wang, Qingyun Li, Xin Li

**Affiliations:** 1 College of Horticulture, Hebei Agricultural University, Baoding 310007, China; wangqianying717@163.com (Q.W.); smy18333897665@163.com (M.S.); 2 Key Laboratory of Tea Quality and Safety Control, Ministry of Agriculture and Rural Affairs, Tea Research Institute, Chinese Academy of Agricultural Sciences, Hangzhou 310008, China; jingboyu625@163.com (J.Y.); hhhawphhhawp@163.com (W.L.); 15225722895@163.com (W.W.); 17863935460@163.com (R.M.); geshibei@tricaas.com (S.G.); aq.mohamed@nrc.sci.eg (A.S.M.); wangly@tricaas.com (L.W.); 3 Nanping Agriculture and Rural Bureau, Nanping 353199, China; 4 College of Horticulture and Plant Protection, Henan University of Science and Technology, Luoyang 471023, China; 5 Horticultural Crops Technology Department, Agricultural and Biological Research Institute, National Research Centre, Dokki, Giza 12622, Egypt

**Keywords:** abiotic stress, biosynthesis, *Camellia sinensis* L., climate change, L-theanine, molecular mechanism, transport

## Abstract

L-theanine, a unique non-protein amino acid predominantly found in tea plants (*Camellia sinensis*), plays a pivotal role in plant responses to abiotic stress and significantly influences tea quality. In this review, the metabolism and transport mechanisms of L-theanine are comprehensively discussed, highlighting its spatial distribution in tea plants, where it is most abundant in young leaves and less so in roots, stems, and older leaves. The biosynthesis of L-theanine occurs through the enzymatic conversion of glutamate and ethylamine, catalyzed by theanine synthase, primarily in the roots, from where it is transported to aerial parts of the plant for further catabolism. Environmental factors such as temperature, light, drought, elevated CO_2_, nutrient unavailability, and heavy metals significantly affect theanine biosynthesis and hydrolysis, with plant hormones and transcription factors playing crucial regulatory roles. Furthermore, it has been demonstrated that applying L-theanine exogenously improves other crops’ resistance to a range of abiotic stresses, suggesting its potential utility in improving crop resilience amid climate change. This review aims to elucidate the physiological mechanisms and biological functions of L-theanine metabolism under stress conditions, providing a theoretical foundation for enhancing tea quality and stress resistance in tea cultivation.

## 1. Introduction

Tea (*Camellia sinensis*), as an important beverage cash crop, is renowned globally for its unique taste and nutritional value [1]. Commercial tea cultivation spans over 50 countries (China, India, Kenya, Sri Lanka, Turkey, Vietnam, and Indonesia lead as the top producers) [2]. Occupying 5 million hectares of land, tea plantations are predominantly found in China, which leads as the major producer with an annual output of 2.5 million tons. Following China are India (1.3 million tons), Kenya, and Sri Lanka. Together, these four nations account for 86% of the total global tea production [3]. Different types of tea possess distinct properties and benefits. Black tea, which is fully oxidized, has a bright red color and a rich aroma. It is known for its antioxidant properties, as well as its ability to warm the stomach, protect the liver, and safeguard cardiovascular health. Green tea, made from slightly oxidized leaves, is rich in amino acids and various vitamins. It offers anti-inflammatory effects, enhances immune function, and improves mental clarity. White tea, consisting of young leaves that have not yet been oxidized, retains the natural substances of fresh leaves and is associated with antioxidant, anticancer, and lipid-lowering effects [4,5]. L-theanine, a non-protein amino acid in tea, significantly influences tea flavor and plays a vital role in enhancing tea quality and promoting human health [6]. Clinical studies have demonstrated that L-theanine can enhance immune function, prevent influenza, and facilitate postoperative recovery. Extensive animal research has further revealed that L-theanine exerts immunomodulatory effects in inflammation, gastrointestinal health, and cancer prevention. It achieves this by boosting the body’s antioxidant capacity, promoting the synthesis of reduced glutathione (GSH). Additionally, L-theanine has been shown to increase stress resistance and immune function in animals. Moreover, L-theanine has been found to alleviate anxiety, lower blood pressure, improve cognitive function, and help relieve stress [7,8]. However, tea plants inevitably face various abiotic stresses, such as drought, salt stress, high as well as low temperature, light exposure, elevated CO_2_ concentration, and heavy metal contamination, which severely constrain tea production and quality [9]. In recent years, the escalating effects of global climate change and various environmental concerns have intensified abiotic stresses on the growth of tea plants, presenting unprecedented challenges for the tea industry [10]. To address these challenges, researchers have increasingly focused on the responses and mechanisms of tea plants to abiotic stresses, with particular attention being paid to the role of L-theanine metabolism [11]. The L-theanine metabolism is closely linked to stress resistance in tea plants and may serve as a strategy for adapting to environmental changes.

Initial studies primarily focused on the synthesis pathways of L-theanine, the identification of metabolic enzymes, and the regulation of gene expression [12,13]. However, the detailed mechanism by which the L-theanine metabolism is regulated in response to abiotic stress remains poorly understood. Recently, the development of molecular biology technology has enabled researchers to explore the relationship between the L-theanine metabolism and abiotic stress through genomics, transcriptomics, and metabolomics [14,15]. Numerous studies have confirmed that there are significant differences in theanine content among different tea plant varieties. These differences affect the quality of tea and are closely related to the stress resistance of tea plants. The theanine content is significantly positively correlated with the taste quality of tea, with high-theanine-content teas typically exhibiting superior freshness, such as the well-known albino mutant (Anji Baicha) [16]. Albino tea cultivars, characterized by their yellow or white leaf variations, were found to possess high concentrations of theanine [16]. Tea plant varieties with high theanine content show stronger stress resistance when facing adverse conditions. The current review emphasizes several key aspects related to the L-theanine metabolism under abiotic stress. First, it will discuss the changes in the synthesis, hydrolysis, and transport pathways of L-theanine in response to various abiotic stressors, including temperature fluctuations, moisture levels, salt stress, carbon dioxide concentration, light intensity, nutrient unavailability, and heavy metal exposure. Additionally, it will explore the regulation of key enzymes and genes involved in L-theanine metabolism during stress conditions, highlighting the roles of hormones and transcription factors in these processes. Furthermore, it will discuss the applications of L-theanine in other crops, highlighting its biological functions and potential benefits. Finally, we outline future research directions that could enhance our understanding of L-theanine’s role in plant stress responses and its applications in agriculture. This review aims to provide new perspectives on the resistance of tea plants to abiotic stress and to establish a theoretical foundation for understanding the response mechanisms of the L-theanine metabolism under abiotic stress, thereby fostering further research in this field.

## 2. Metabolism and Transport of L-Theanine in Tea Plants

### 2.1. Spatial Distribution of L-Theanine in Tea Plants

L-theanine, as a unique amino acid, is widely distributed across various organs of tea plants, excluding the fruits [6,17,18,19]. As theanine is predominantly concentrated in root and young leaf tissues, the accumulation in stems and flowers is significantly lower than that of roots and young leaves, as determined by the analysis of L-theanine subcellular localization [20,21,22]. The dynamic characteristics of L-theanine metabolism in tea plants was further confirmed in recent studies by using matrix-assisted laser desorption/ionization mass spectrometry imaging (MALDI-MSI) for spatial analysis of various tissues [23]. In the new shoots of tea plants, L-theanine constitutes 50–70% of the total free amino acid content, but the proportion of L-theanine decreases progressively with leaf aging [24,25]. This decrease occurs because L-theanine is hydrolyzed into compounds like catechins and tea polyphenols during shoot growth [26,27]. It is vital to explore the L-theanine spatial distribution, which could benefit the optimization of cultivation practices and improvements in tea quality.

### 2.2. Biosynthesis of L-Theanine in Tea Plants

As a glutamine analog, L-theanine is biosynthesized from glutamate and ethylamine catalyzed by *Camellia sinensis* theanine synthase (CsTS) [28]. Among them, glutamate as a key precursor for L-theanine synthesis, is primarily produced in tea plants roots via amino acid metabolic pathways that utilize the soil nitrogen and involve the enzymes such as *Camellia sinensis* glutamine synthetase (CsGS), *Camellia sinensis* Arginine Decarboxylase (CsADC), *Camellia sinensis* glutamate synthase (CsGOGAT), and *Camellia sinensis* glutamate dehydrogenase (CsGDH) [29,30]. Ethylamine is generated via alanine decarboxylation catalyzed by *Camellia sinensis* alanine decarboxylase (CsAlaDC), and its content directly determines the accumulation level of L-theanine [31,32,33]. Thus, the uniquely present and available ethylamine in tea plants plays a critical role in L-theanine synthesis. While the TS demonstrates high specificity for glutamate and ethylamine, positively regulating the L-theanine accumulation in tea leaves [22,26].

Previous studies have confirmed that L-theanine is predominantly synthesized in the roots of tea plants [30,34,35]. Zhang et al. sequenced tea plants’ transcriptome using PacBio SMRT technology and found that key genes for L-theanine synthesis, including *CsTSI* (which synthesizes glutamate and ethylamine into theanine), *CsADC* (which catalyzes the decarboxylation of arginine to produce putrescine and thereby participates in the synthesis of polyamines), and *CsGOGAT* (which catalyzes ammonium assimilation and produces the precursor glutamate for theanine production), are highly expressed in the root system, which further supports the root-based synthesis of L-theanine [36]. Additionally, the high expression of the *Camellia sinensis* Alanine Aminotransferase (*CsALT*) gene in the roots facilitates the conversion of ammonia into ethylamine, a critical precursor of L-theanine biosynthesis, promoting its accumulation [37]. Subcellular localization and gene expression analyses identified the cytosol of root cells as the primary site for theanine biosynthesis, with *CsTSI* specifically expressed in the roots [22]. Moreover, the presence of subcellular components of L-theanine in the cytosol and chloroplasts of tea buds indicates that a small amount of L-theanine is also synthesized in the leaves of tea plants, although the concentration is relatively lower compared to that in the roots [37].

### 2.3. Hydrolysis of L-Theanine in Tea Plants

L-theanine synthesized in the roots of tea plants is transported to above-ground parts, where it is catabolized in the leaves [24]. L-theanine hydrolysis, similar to other amino acids, involves deamidation and deglutamination to produce glutamate and ethylamine, catalyzed by L-theanine hydrolase [38]. These two products subsequently participate in the other metabolic pathways for reuse. For instance, it was found that the degradation products of L-theanine serve as substrates for the synthesis of other polyphenols and amino acids [39]. Glutamate primarily contributes to nitrogen-containing compound synthesis or further hydrolysis, while ethylamine is oxidized to acetaldehyde for the biosynthesis of catechins [26]. Additionally, L-theanine degradation depends on specific light conditions. It was found that γ-glutamyl transpeptidase (CsGGT2) exhibits high catalytic efficiency in the degradation of L-theanine, with this process being regulated by light [40]. Additionally, the pyridoxine biosynthesis enzyme, CsPDX, has been demonstrated to catalyze the hydrolysis of L-theanine into glutamate and ethylamine in vitro [41]. Deng et al. speculated that L-theanine degradation capacity in the young tea leaves is lower than that of mature leaves, potentially explaining their weaker photosynthetic ability [35]. In conclusion, the hydrolysis process is essential in L-theanine metabolism, and it plays a crucial role in maintaining metabolic fluxes to various processes [39].

### 2.4. Transportation of L-Theanine in Tea Plants

**Figure 1:**
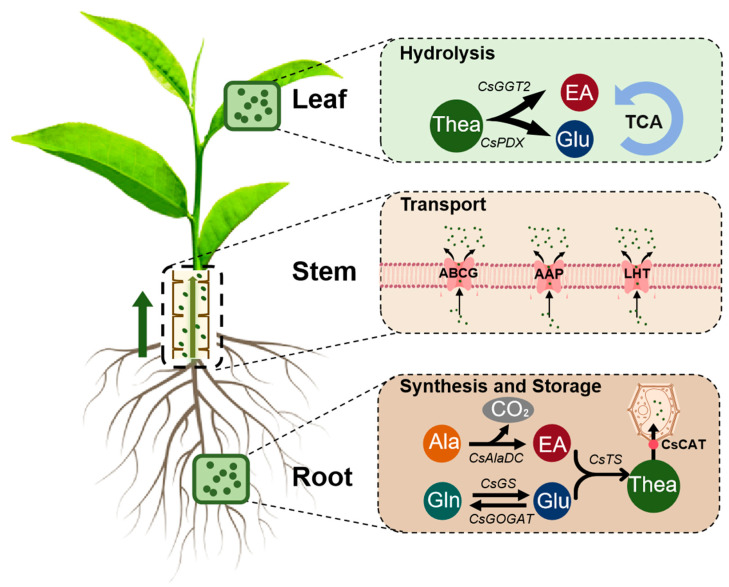
Metabolism and transport of theanine in tea plants. The roots of the tea plant are the primary site for synthesizing and storing theanine. Theanine is then transported upwards through transport proteins. Theanine is catabolized to Glu and EA in the shoots. [Thea, theanine; Glu, glutamate; Ala, alanine; Gln, glutamine; EA, ethylamine; TCA, tricarboxylic acid cycle; CO_2_, Carbon dioxide; *CsGGT2*, *Camellia sinensis* γ-glutamyl-transpeptidase; AAP, amino acid permease; ABCG, ATP-binding cassette sub-family G; LHT, lysine-histidine-like transporter; CsCAT, *Camellia sinensis* cationic amino acid transporter; *CsPDX*, *Camellia sinensis* pyridoxine biosynthesis; *CsTS*, *Camellia sinensis* theanine synthetase; *CsAlaDC*, *Camellia sinensis* alanine decarboxylase; *CsGS*, *Camellia sinensis* glutamine synthetase; *CsGOGAT*, *Camellia sinensis* glutamine-2-oxoglutarate aminotransferase].

The transport of L-theanine in tea plants is a complex and critical process that ensures its distribution throughout the plant. As it is synthetized in the root, L-theanine is transported through the xylem, primarily accumulating in young leaves, to regulate the physiological activities in the tea plant [42]. This transport of L-theanine in tea plants relies primarily on the combined effects of the water potential gradient from transpiration and the plant’s pressure flow mechanism [30]. Meanwhile, the substantial synthesis of L-theanine in the roots indirectly facilitates this transport process. As a mobile nitrogenous compound, L-theanine is transported from roots to shoots and is redistributed among organs based on growth demands [43]. During vigorous bud growth, L-theanine is reallocated from older leaves to new buds, preferentially accumulating in young tissues [21]. Transport proteins play an indispensable role in L-theanine’s inter-organ transport. Through the study of the correlation between the expression of transporter genes and the content of L-theanine, Li et al. found that Lysine-histidine-like transporter (CsLHT), as an amino acid transporter, can positively regulate the content of L-theanine and possesses the capability to transport L-theanine [44,45]. Similarly, Amino acid permeases (AAPs), highly expressed in roots and stems, are confirmed to transport L-theanine [46]. Cationic amino acid transporter (CsCAT2), a vacuolar transporter protein with a moderate affinity for L-theanine, is specifically expressed in roots and stems [47,48]. The ATP-binding cassette transporter (CsABCG11.2) simultaneously transports cadmium and L-theanine, aiding in cadmium toxicity alleviation [49]. This implies that L-theanine transport proteins also play a critical role in reallocating L-theanine in response to environmental stresses (Figure 1).

## 3. Response of L-Theanine Metabolism to Stress in Tea Plants

### 3.1. Effect of Temperature Stress on L-Theanine Metabolism

Recent studies have revealed that temperature changes significantly affect the theanine metabolism in tea plants [50,51]. Some studies indicate that summer tea grown in high temperatures produces a more bitter tea with lower L-theanine content, whereas cool weather increases L-theanine levels [44]. Temperature stress affects L-theanine synthesis, hydrolysis, and transport.

Also, some studies indicate that a temperature range of 15–25 °C is conducive to the synthesis of theanine [52]. High and low temperatures adversely affect L-theanine biosynthesis. At 38 °C, the activity and expression of L-theanine synthesis enzymes significantly decrease [53]. Meanwhile, high temperatures degrade key synthetic enzymes like GOGAT and GS, downregulate L-theanine biosynthetic genes, and reduce glutamate levels, weakening L-theanine synthesis [54,55]. Additionally, Wang et al. observed that low temperatures reduce the proliferation of young shoots and buds and inhibit metabolic enzyme activity, with inhibition lessening as temperature rises [56]. At low temperatures, the expression of *CsFd-GOGAT*, which is negatively correlated with L-theanine content, increases, leading to a reduction in L-theanine accumulation [54]. These findings confirm that temperature stress adversely affects L-theanine biosynthesis.

Temperature significantly impacts L-theanine hydrolysis catabolism. High temperatures lead to an accumulation of nitrogen-containing compounds in tea plants, which stimulates the activity of hydrolase enzymes and accelerates the hydrolysis of L-theanine, thereby resulting in a reduction in its content [53]. Studies on phenol–ammonia ratios reveal that high temperatures accelerate L-theanine degradation into tea polyphenols [57]. Moreover, Chen et al. observed that the higher temperature in late spring increases *CsGDHs* expression, accelerating L-theanine decomposition. The CsPDX2.1 protein (pyridoxal 5′-phosphate synthase subunit), a confirmed L-theanine hydrolase, is also temperature-sensitive [58]. Fu et al. found that CsPDX2.1 hydrolase shows peak activity at 35–40 °C in enzyme reactions across six temperatures. When the temperature exceeds this range, enzyme activity declines sharply, slowing down L-theanine catabolism [41]. These findings indicate that hydrolysis of L-theanine is temperature-regulated.

L-theanine transport also responds to temperature changes. Ruan et al. found that L-theanine transport from roots to buds is influenced by seasonal temperature variations, being weakest during the summer and winter months, with the strongest transport in early spring [59]. L-theanine transport relies on transport proteins, whose expression is temperature-sensitive. For instance, CsAAPs, a family of L-theanine transport proteins, show seasonal temperature-regulated expression, with lower *CsAAP1* and *CsAAP2* levels in winter and upregulation in spring [30]. Some studies on *CsLHT* confirm that its expression aligns with changes in L-theanine content in tea [44]. The expression pattern of *CsCAT2* in the roots aligns closely with the storage trend of L-theanine in the roots across various time points from winter to spring [48].

### 3.2. Effect of Light on L-Theanine Metabolism

Light is one of the important meteorological factors affecting the growth and quality of tea plants [60]. Light intensity and quality affect photoreceptors and signaling pathways, ultimately regulating plant growth, development, and secondary metabolism [29]. Notably, the L-theanine metabolism is regulated by light conditions. Particularly, L-theanine hydrolysis into glutamate and ethylamine is light-dependent, and thus sunlight intensity negatively affects L-theanine accumulation [61,62]. Reducing light exposure is an effective method to increase theanine content [15,58]. Therefore, shading is commonly used to promote theanine accumulation in young tea shoots [50,63,64]. Yeast two-hybrid studies show that shading, compared to natural light, significantly increases ethylamine content, enhances biosynthetic enzyme activity, and induces the expression of key genes (*CsGOGAT*, *CsAlaDC*, *CsTSI*, and *CsGS1.1*), thereby stimulating theanine synthesis in tea roots [65]. Additionally, shading promotes theanine allocation between young leaves and tender stems by regulating AAP transport protein expression, increasing accumulation in tender stems. Dark treatment indirectly downregulates ELONGATED HYPOCOTYL5 (*HY*5) expression, reducing *CsGGT*2-mediated theanine hydrolysis and maintaining theanine levels [40]. In albino tea leaves, theanine hydrolysis is suppressed compared to normal leaves, resulting in higher theanine levels [66,67].

Different light qualities also influence theanine metabolism. Red light induces the expression of amino acid metabolism genes, including *CsGDH*, linked to theanine biosynthesis, as observed in the photosensitive tea cultivar ‘Huangjinya’ under red light treatment [68]. Theanine metabolism relies on glutamine, the red light treatment promotes the accumulation of N-a-acetyl-L-glutamine and L-asparagine [56]. Transcriptional analysis showed that red light induces differential expression of the Glutathione S-transferase (*GST*) family genes. Moreover, red light enrichment alters the expression of amino acid transport and metabolism genes, including the amino acid Trans ELONGATED HYPOCOTYL5 porter (*CAATs*) and L-cysteine desulfurase (*CDH*). These findings strongly suggest that red light influences theanine content. Red light treatment likely promotes theanine accumulation by inducing the expression of precursor substances, theanine-related genes, and transporter proteins, although the exact mechanism needs further study. RNA-seq analysis revealed that amino acid synthesis in the tea plant is significantly dependent on blue light compared to purple, yellow, and natural light treatments [69].

### 3.3. Effect of Drought Stress and Salinity on L-Theanine Metabolism

Tea plants thrive in moist conditions, while drought, often coupled with high temperatures, inhibits theanine accumulation [70]. Water deficiency suppresses the expression of theanine biosynthesis genes (*CsGOGAT*, *CsGDH*, *CsAlaDC*, and *CsTSI*) [55,71,72,73]. Water deficit signals activate the key enzyme gene theanine hydrolase (*ThYD)* in the theanine biodegradation pathway, inducing theanine hydrolysis. Similar findings show that water loss triggers L-theanine hydrolysis, reducing its accumulation in tea leaves [74]. Cheng et al. modeled three water content levels and found that water deficit stimulates *CsPDX*2.1 expression, indicating its role in reducing theanine content [75]. In summary, theanine responds to drought stress by altering the expression levels of genes related to biosynthesis and biodegradation, resulting in a decrease in theanine content.

The content of theanine in tea plants was significantly induced by the environmental stress of salt stress. It was found that salt treatment stimulated the expression of *CsTS* and promoted the synthesis of theanine in tea plants [43]. Recent research has likewise shown that the salt stress induces the expression of theanine synthesis genes. The report found that the contents of alanine, glutamate, glutamine, and GABA, which are highly related to theanine synthesis pathways, were significantly increased when tea plants were exposed to the nutrient solution of NaCl for 3 days [76]. This highlights the remarkable functionality of theanine in response to environmental stresses. The accumulation of theanine, a metabolic product, contributes to stabilizing and maintaining the quality level of tea under adverse conditions, thereby enhancing the stress resistance of tea plants.

### 3.4. Effect of Elevated CO_2_ Concentration on L-Theanine Metabolism

Rising CO_2_ levels, driven by global climate change, directly impact the growth and theanine metabolism in tea plants. Elevated atmospheric CO_2_ promotes photosynthesis, influencing the plant carbon-nitrogen balance [77]. In terms of primary metabolism, enhanced photosynthesis aids photosynthetic product accumulation and key metabolite regulation, supplying precursors for theanine synthesis; elevated CO₂ levels boost the respiration rates and significantly increase biomass accumulation in tea plants [78]. High CO_2_ concentrations increase theanine content in tea leaves and upregulate theanine biosynthesis genes (*CsGS* and *CsTS*) [11,79]. Similar findings suggest that 24–60 days of high CO_2_ exposure significantly increases theanine levels, likely due to improved nitrogen utilization and a higher carbon-nitrogen ratio, thus enhancing tea quality [80,81]. In addition, the high concentration of CO_2_ can mitigate the negative effects of high temperatures and alleviate the photosynthetic metabolism of tea plants under heat stress, maintaining a certain level of theanine synthesis capacity [82]. Therefore, increasing CO_2_ levels is a great strategy that holds promise for enhancing theanine content in tea plants.

### 3.5. Effect of Nutrient Elements and Heavy Metal Stress on L-Theanine Metabolism

Disparities in nitrogen, phosphorus, potassium, and magnesium levels alter theanine synthesis, transport, and accumulation, impacting tea quality [83]. Nitrogen plays a crucial role in theanine metabolism [20,84]. Adequate nitrogen fertilizer significantly increases theanine content in fresh tea leaves [85]. Nitrogen deficiency affects theanine accumulation through multiple pathways [86,87]. Low nitrogen stress reduces glutamate and ethylamine supply, essential for theanine synthesis. Low nitrogen stress downregulates theanine synthesis genes and upregulates *CsAAPs*, promoting theanine transport [21,88,89,90]. In addition, excess nitrogen also disrupts theanine metabolism. A report found that excessive nitrogen shifts the metabolism toward other nitrogen-containing compounds, increasing allocation to free amino acids and reducing theanine accumulation [91]. Moreover, nitrogen forms affect theanine metabolism [20]. NH_4_^+^ nitrogen significantly enhances nitrogen allocation to theanine compared to NO^3−^, which is less efficiently assimilated [92]. The difference in assimilation likely arises from the form of nitrogen that influences enzyme activity [93]. Furthermore, NH_4_^+^ supplementation increases GS activity, favoring substrate synthesis in roots [91]. This evidence emphasizes the importance of N form in regulating theanine metabolism in tea.

Magnesium plays an important role in the metabolism of theanine [82]. Adequate Mg supply promotes theanine synthesis in roots and accumulation in young buds [59]. Mg deficiency disrupts the transcription of theanine biosynthesis genes, alters compound distribution in the above-ground and under-ground tissues, and inhibits theanine accumulation [94,95,96]. In addition, low phosphorus and potassium levels inhibit theanine synthase activity, thereby reducing theanine synthesis and affecting tea plant physiology [83,97,98,99,100,101]. Theanine content is positively regulated within optimal phosphorus and potassium levels, but excessive amounts inhibit accumulation [102,103]. Manganese can promote the accumulation of theanine due to its involvement in the activation of the nitrate reduction process; molybdenum can increase the intake and utilization of nitrogen fertilizer, so the deficiency in molybdenum affects the synthesis of theanine in tea plants [104].

**Figure 2:**
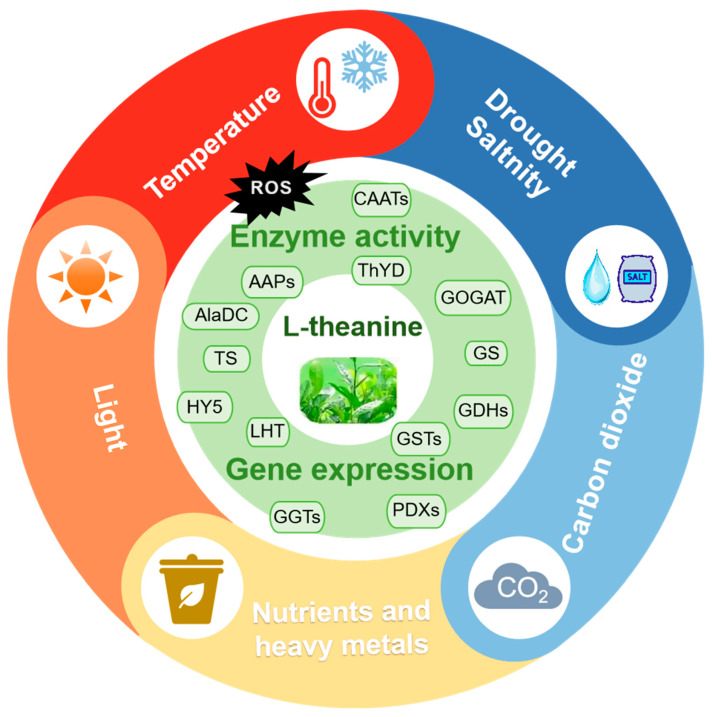
Metabolism of theanine as influenced by environmental factors. Theanine metabolism is affected by abiotic stresses such as temperature, light, drought, and carbon dioxide concentration, as well as nutrient elements and heavy metal stresses. This is manifested by the production of reactive oxygen species (ROS), changes in enzyme activity, and alterations in gene expression. ROS, reactive oxygen species; *CAATs*, amino acid transporters; *AAPs*, amino acid permease; *GOGAT*, glutamine-2-oxoglutarate aminotransferase; *AlaDC*, alanine decarboxylase; *GS*, glutamine synthetase; *TS*, theanine synthetase; *LHT*, lysine-histidine-like transporter; *GGTs*, γ-glutamyl-transpeptidases; *HY5*, ELONGATED HYPOCOTYL 5; *GDHs*, glutamate dehydrogenases; *PDXs*, pyridoxine bio-synthesists; *GSTs*, Glutathione S-transferases; *ThYD*, theanine hydrolase.

Heavy metal exposure disrupts theanine metabolic pathways, altering its concentration in tea plants [105,106]. Heavy metals such as cadmium (Cd), Chromium (Cr), and lead (Pb) reduce chlorophyll content and suppress photosynthetic and respiratory rates, which in turn inhibits the accumulation of dry matter in the roots, stems, and leaves of tea plants [107]. Recently, a study has found that, under Pb and Cd stress, the content of caffeine and free amino acids decreased significantly in tea plants, which may be the reason for the weakening of nitrogen metabolism [108]. Moreover, aluminum (Al), manganese (Mn), and copper (Cu) in tea leaves trigger oxidative stress and inhibit the activity of enzymes involved in theanine biosynthesis, disrupting its metabolism and promoting degradation [109,110]. It is worth noting that, in tea plants, contradictory results show that aluminum can inhibit the biosynthesis of theanine, which contributes to the flavor of green tea [111]. However, aluminum treatment can also increase the amount of theanine in tea plant leaves and roots [112]. Heavy metals alter the expression of key genes regulating the theanine metabolism [113]. Additionally, oxidative stress induced by heavy metals in tea plants at the cellular level can lead to the degradation of theanine and other amino acids, further affecting the quality of tea products [114,115] (Figure 2).

## 4. Molecular Mechanism of L-Theanine Metabolism to Stress in Tea Plants

### 4.1. Effect of Plant Hormones and Growth Regulators on L-Theanine Metabolism

Abiotic stress affects the growth, development, and quality of tea. Tea plants respond to stress by activating adaptive mechanisms to environmental changes. Plant hormones, as endogenous signaling molecules, are critical in helping tea plants cope with stress [116,117,118]. Plant growth regulators and hormones enhance the tea plant’s resistance and quality by regulating theanine metabolism under stress. Foliar application of glycine betaine significantly increases theanine content under low temperatures [119]. Exogenous brassinosteroid (BR) and melatonin mitigate high-temperature-induced inhibition of theanine accumulation [116,117,118]. The brassinosteroid upregulates transcription of *CsADC*, *CsGS*, *CsGOGAT*, and *CsTS*, similar to melatonin effects [120,121]. Moreover, the activity of *ADC* is enhanced by BR to promote ethylamine synthesis, thereby improving theanine metabolism. Similarly, jasmonic acid (JA), jasmonic acid-isoleucine (JA-Ile), and methyl jasmonate (MeJA) were found to be closely related to theanine content, with carbonic anhydrase (CsCA) positively regulating the synthesis of theanine, while glutamate decarboxylase (CsgadB) negatively affects theanine accumulation. Hormones such as JA collectively upregulate the expression of *CsCA* and downregulate the expression of *CsgadB*, improving theanine imbalance caused by temperature changes. Some studies found a positive correlation between JA and theanine content [122]. Furthermore, exogenous gibberellic acid (GA) enhances theanine biosynthesis, increasing production by 27%, as confirmed by seasonal GA dynamics during winter dormancy [123]. Additionally, the synthesis of abscisic acid (ABA) influences the activation of Abscisic acid responsive element-binding factors (*CsABF7)*, which in turn induces *CsWRKY40* expression, leading to the hydrolysis of L-theanine during the withering process of tea leaves [124]. Additionally, metabolomic analysis indicated that the inhibition of ABA treatment stimulates the differential expression of genes encoding nitrate reductase (NR) and nitrate transporter (NRT), promoting the accumulation of theanine [125]. These findings collectively demonstrate that ABA negatively regulates the accumulation of theanine. Salicylic acid (SA) regulates theanine metabolism in tea seedlings in a concentration-dependent manner, with appropriate SA levels enhancing theanine synthesis [126].

### 4.2. Effect of Transcription Factors on L-Theanine Metabolism

Transcription factors (TFs) are key regulators of gene expression and play a critical role in the response of theanine metabolism to stress [127,128,129]. Abiotic stress triggers cellular signaling in tea plants, regulating the expression of *WRKY* domain-containing transcription factors. CsWRKY responds to abiotic stress through multiple pathways [127]. Drought stress stimulates *CsWRKY40* expression during water loss from tea leaves and withering. A weighted gene co-expression network analysis revealed its activation of the *CsPDX2.1* promoter, regulating L-theanine hydrolysis. WRKY also regulates the ABA signaling pathway to mediate abiotic stress responses. Similar studies show that JA and other hormones mitigate high-temperature inhibition of theanine metabolism by modulating CsWRKY activity, with jasmonate ZIM-domain (JAZ) family of proteins interacting with *WRKY22* [75,128]. In addition, MYB transcription factors broadly regulate secondary metabolism in tea plants, with abiotic stress or hormone treatment significantly activating *MYB* gene expression [130]. CsMYB42, an R2R3-MYB transcriptional activator, promotes *CsGS1* expression, regulating theanine synthesis [131,132]. CsMYB73, a nuclear transcriptional repressor, inhibits *CsGS* and *CsGGT* expression, regulating theanine hydrolysis in response to seasonal temperature stress [133]. CsMYB6 activates *CsTSI* transcription to regulate theanine metabolism, while CsMYB40 binds to *CsAlaDC*, enhancing theanine synthesis [76]. In addition, under drought stress, *CsMOF1* binds to the *CsGS1* promoter, thereby downregulating *CsGS1* expression and inhibiting theanine accumulation [134]. Tea lateral organ boundaries domain (*CsLBD37*) regulates theanine synthesis and *CsAlaDC* expression. Despite its high correlation with *CsAlaDC*, *CsLBD37* inhibits its expression [76]. Furthermore, *Camellia sinensis* C-repeat binding factor 4 (*CsCBF4*) binds to *CsAlaDC*, which facilitates ethylamine accumulation and mediates osmotic stress responses by maintaining reactive oxygen species homeostasis [135]. Under light stress, *CsHY5* expression is upregulated, activating *CsGGT2* and promoting theanine hydrolysis. Conversely, darkness suppresses CsHY5 transcription, increasing theanine accumulation, indicating that HY5 negatively regulates amino acid levels [40]. The transcriptional regulatory network of theanine metabolism is intricate, involving potential interactions among transcription factors that regulate gene expression through a multi-level multi-pathway system [136].

## 5. Application of L-Theanine in Abiotic Stress Tolerance

**Figure 3:**
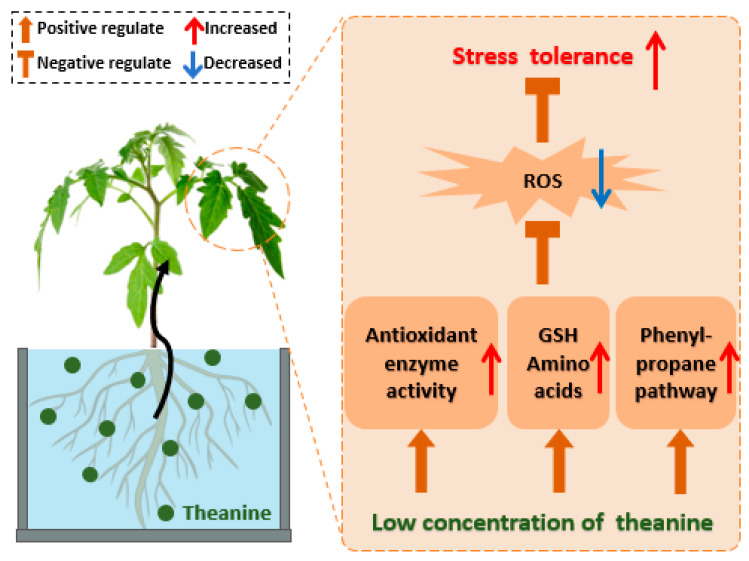
The application of theanine in other crops. Theanine plays a key role in plant production by enhancing stress tolerance. This is specifically manifested in enhancing the activity of antioxidant enzymes, promoting the production of glutathione, facilitating the conversion of other amino acids, and stimulating the phenyl–propanoid pathway. Low concentrations of theanine can reduce the accumulation of reactive oxygen species (ROS), thereby significantly enhancing the stress resistance of crops [GSH, glutathione].

Theanine plays a key role in plant production by enhancing both plant quality and stress resistance. Theanine regulates redox homeostasis in tea plants, boosting antioxidant enzyme activity and gene expression and significantly enhancing salt stress tolerance [137]. Similar studies have also confirmed that theanine can help tobacco resist drought stress and low potassium stress [138]. Treatment with 0.1 and 0.2 mmol·L^−1^ theanine increases the reduced glutathione (GSH) and proline content in tobacco seedlings, enhancing antioxidant enzyme activity and alleviating oxidative damage. Theanine promotes potassium uptake in tobacco seedlings by inducing potassium absorption gene expression. Similarly, 0.25 mmol·L^−1^ of theanine enhances the resistance of tobacco seedlings to drought stress (Figure 3). Additionally, theanine also influences plant growth and development in a dose-dependent manner. A previous study found that low concentrations of theanine promote tobacco seedling growth, increasing fresh weight, root length, and chlorophyll content [138]. However, other studies show that high concentrations of theanine disrupt the antioxidant system in tobacco seedlings, causing lipid oxidation and oxidative damage, thus inhibiting growth. Yan et al. indicated that the 0.1 mM theanine as spray treatment on the albino the tea plant ‘Huangjinya’ resulted in a 10.1% increase in bud weight [139]. Furthermore, the application of 1 mM theanine significantly induced the growth of new buds, thereby enhancing the yield by 25.4%. Concurrently, the 1 mM theanine spray significantly increased the ratio of auxin (IAA) to abscisic acid (ABA) in the new buds. These findings suggest that exogenous supplementation with theanine holds the potential to enhance both crop yield and aroma quality. This indicates that the application of theanine may facilitate the dual improvement of tea plant’s yield and the sensory attributes of tea, thereby laying the groundwork for further research and potential practical applications.

Theanine metabolism in plants involves the transformation of amino acids, with glutamate and γ-aminobutyric acid (GABA) being key products [78,140]. These transformations affect the overall metabolism and play a role in plants’ responses to environmental stress. Glutamate is a key amino acid in plants, serving as a precursor for protein synthesis, nitrogen metabolism, and disease-related secondary metabolites [141]. Glutamate also is converted into other amino acids like proline and arginine, which are crucial for plant stress responses [142,143]. For instance, glutamate stimulated the growth of tomatoes and increase the density of bacterial populations, thereby mitigating the damage caused by salt stress [144]. By applying exogenous glutamate, the chlorophyll contents in Chinese cabbage (*Brassica rapa* L. ssp. *pekinensis*) can be elevated, which in turn stimulates growth. Additionally, the exogenous glutamate treatment prevents the loss of electrolytes and the build-up of reactive oxygen species, thereby enhancing the heat resistance of Chinese cabbage [145]. Similarly, GABA is essential in plants’ responses to environmental pressures [146,147]. GABA regulates osmotic pressure and redox balance in cells of sunflowers (*Helianthus annuus* L.), tomatoes (*Solanum lycopersicum* L.), and common andrographis herb (*Andrographis paniculata*), helping plants withstand abiotic stress [148,149,150]. Additionally, several studies provided a comprehensive review of the application of exogenous GABA in enhancing plants’ tolerance to stress [151,152]. It elaborates on the multifaceted mechanisms by which GABA achieves this effect, including inducing the production of Nitric Oxide (NO), activating enzymes involved in stress responses, enhancing the activity of antioxidants, and strengthening membrane stability, among other pathways.

## 6. Prospects and Conclusions

Tea is an important agricultural crop with special cultural significance and is one of the most widely consumed beverages globally. The tea industry has a profound impact on the local economy, driving the economic development of many regions (such as China, India, and Kenya) and making a significant contribution to international trade. In addition, with the growing demand for high-quality tea, it has become particularly important to enhance the plant’s resistance to environmental stress. Tea was selected as the test plant and has direct significance for improving crop yield, quality, and sustainability, thereby benefiting the agricultural sector and the overall economy.

Currently, theanine plays an important role in the resistance of tea plants to abiotic stress. Future research should investigate the specific mechanisms by which theanine influences signaling pathways and clarify its role in the stress response network. A deeper understanding of the theanine signaling pathway could lead to the development of biotechnological tools to regulate its metabolism via genetic engineering or agronomic practices, enhancing the resilience of tea plants and other crops. Enzymes and genes involved in theanine synthesis and hydrolysis pathways are promising targets for genetic engineering. Gene editing technology can target and modify these enzymes and genes to enhance tea plant’s tolerance to stress. In addition, studying the regulatory mechanisms of key enzymes in theanine metabolism will aid in developing novel bioregulators to improve crop yield and quality. Furthermore, as a potential bioactive substance, theanine may have a promising application in agricultural production. Exogenous application of theanine or its precursors can serve as a novel plant growth regulator, enhancing stress tolerance and crop yield. In addition, the role of theanine in enhancing crop disease resistance provides a theoretical basis for the development of novel and environmentally friendly plant protection strategies.

In conclusion, this review on the response mechanism of theanine metabolism to abiotic stress offers new insights into tea plant’s adaptation mechanisms and provides innovative approaches for cultivating tea and other crops. Future research should further explore the signal transduction and metabolic regulation of theanine, as well as its applications in agriculture, to support the sustainable development of the global tea industry.

## Data Availability

The data presented in this study are available on request from the corresponding author. The data are not publicly available due to privacy.
